# Circular RNA profile indicates circular RNA VRK1 is negatively related with breast cancer stem cells

**DOI:** 10.18632/oncotarget.21183

**Published:** 2017-09-23

**Authors:** Ningning Yan, Haiyan Xu, Jinnan Zhang, Liang Xu, Yanyun Zhang, Le Zhang, Yingchun Xu, Fengchun Zhang

**Affiliations:** ^1^ Department of Oncology, Ruijin Hospital, Shanghai Jiaotong University School of Medicine, Shanghai 200025, China; ^2^ Department of Oncology, Suzhou Kowloon Hospital, Shanghai Jiaotong University School of Medicine, Suzhou 215021, China; ^3^ Department of Neurosurgery, China-Japan Union Hospital, Jilin University, Changchun, 130031, China; ^4^ Prevention and Cure Center of Breast Disease, Third Hospital of Nanchang, Nanchang, 330009, China; ^5^ Institute of Health Sciences, Shanghai Institutes for Biological Sciences, Chinese Academy of Sciences & SJTUSM, Shanghai 200031, China; ^6^ Department of Oncology, Renji Hospital, Shanghai Jiaotong University School of Medicine, Shanghai 200127, China

**Keywords:** breast cancer stem cells, circular RNA, RNA-sequencing, *circVRK1*

## Abstract

Circular RNAs (circRNAs), a novel type of noncoding RNAs (ncRNAs), have been shown to be implicated in biological processes including cancer as gene expression regulators. However, the roles of circRNAs in cancer stem cells (CSCs) have been unexplored. In the present study, we screened the circRNA profile in breast cancer stem cells (BCSCs) using RNA-Sequencing. Here, 27 circRNAs were found to be aberrantly expressed. Of these, 19 circRNAs were downregulated and 8 were upregulated and some of these circRNAs were validated by Q-PCR. Furthermore, we constructed the circRNA/miRNA network by bioinformatics approaches and hypothesized that circRNAs might be involved in stemness of BCSCs via serving as miRNA sponges. Importantly, we found that circular RNA VRK1 (circVRK1) could suppress BCSC's expansion and self-renewal capacity. Collectively, the present work provides the first reported evidence of the circRNA profile and circRNA/miRNA interplay in BCSCs. In addition, these findings lay foundation to explore the functions of circRNAs in CSCs and indicate that *circVRK1* might be a promising target for BCSCs.

## INTRODUCTION

Breast cancer is a problem for women worldwide in both developed and underdeveloped countries. According to the statistics, breast cancer is the most common cancer diagnosed and the second leading cause of cancer-related death among U.S. women. On a practical level, it makes up 1 in 3 cancers [1]. It has been reported that even with standard breast cancer treatment, up to 30% of patients still die of relapse or metastasis [2]. Accordingly, identifying the mechanisms underlying breast cancer metastasis and recurrence is the key to eradicate breast cancer.

Currently, accumulating evidence suggests that breast cancer departs from a fraction of cancer initiating cells called cancer stem cells (CSCs) [3–5]. CSCs share with normal stem cells some capacities including self-renewal and pluripotency. Hence, CSCs are considered as the cause of treatment failure and are liable for metastatic dissemination. Conventional therapies, despite target the progeny of CSCs, fail eventually owing to not killing CSCs, which results in recurrence of tumours [5–8]. Therefore, eliminating CSCs might have promise to achieve a permanent cure for breast cancer patients.

In the past few decades, mounting lines of reports have indicated that signaling pathways, including the Notch pathway, Wnt/β-catenin pathway and hedgehog pathway, are responsible for controlling the self-renewal characteristicsof CSCs [9–11]. Conversely, these pathways might contribute to stemness maintenance in CSCs when these pathways become dysregulated. Additionally, an increasing number of recent studies have revealed that non coding RNAs (ncRNAs) are related to stemness of CSCs in a manner of competing for the microRNA response elements (MREs) [12–14]. However, whether circular RNAs (circRNAs), a new type of ncRNAs, are implicated in stemness maintenance of CSCs remains poorly explored.

As a new member of ncRNAs, unlike their linear counterparts, circRNAs are able to form covalent closed circles with their 3′ heads and 5′ tails bonded together [15]. Exactly this special form confers circRNAs resistance to digestion of Rnase R and makes them ideal biomarkers for diagnosis. In addition, circRNAs are reported to be expressed in a tissue/developmental-stage-specific manner [16–18]. Significantly, several reports indicate that circRNAs harbour microRNA (miRNA) binding sites as miRNA sponges [17, 19].

Recently, circRNAs were demonstrated to be involved in abundant biological processes, including cell development, cell proliferation, cancer onset and progression [18, 20–23]. For instance, Simon J. Conn and his colleagues showed that thousands of circRNAs could be produced when cells were underwent epithelial mesenchymal transition (EMT) [21]. In addition, a recent reports revealed that fusion circRNAs generated during chromosomal translocations displayed remarkable abilities to promote cellular transformation *in vitro* and initiate tumours *in vivo*. EMT imparted cancer cells heritable phenotypic changes via epigenetic modifications. Once EMT was activated, cancer cells lost their epithelial characteristics and acquired mesenchymal features and CSC-like characteristics [24, 25]. Hence, these studies promoted us to hypothesize that circRNAs might be implicated in CSC properties. However, reports on circRNAs in CSCs were scarce. Hence, the present work aimed to establish the relationship between CSCs and circRNAs, which might be beneficial for understanding the biogenesis of CSCs.

In this paper, we examined the circRNA signature in breast cancer stem cells (BCSCs) and found 27 differentially expressed circRNAs, of which 19 circRNAs were downregulated and 8 were upregulated. In addition, some representative circRNAs were selected to testify their authenticity by Quantitative PCR (Q-PCR). Gene Ontology (GO) and Kyoto Encyclopedia of Genes and Genomes (KEGG) pathway analysis were used to investigate the possible functions of these circRNAs and a circRNA-miRNA network was constructed to illustrate the potential mechanisms. Specifically, we showed that *circVRK1* could serve as a suppressor on stemness of BCSCs. Collectively, our work provides strong supports for exploring the functional roles of circRNAs in BCSCs. These findings might be beneficial for us to develop new avenues to conquer breast cancer.

## RESULTS

### Mammosphere cells exhibit a much higher percentage of BCSCs with CD44^+^CD24^-^ phenotype

**Figure 1:**
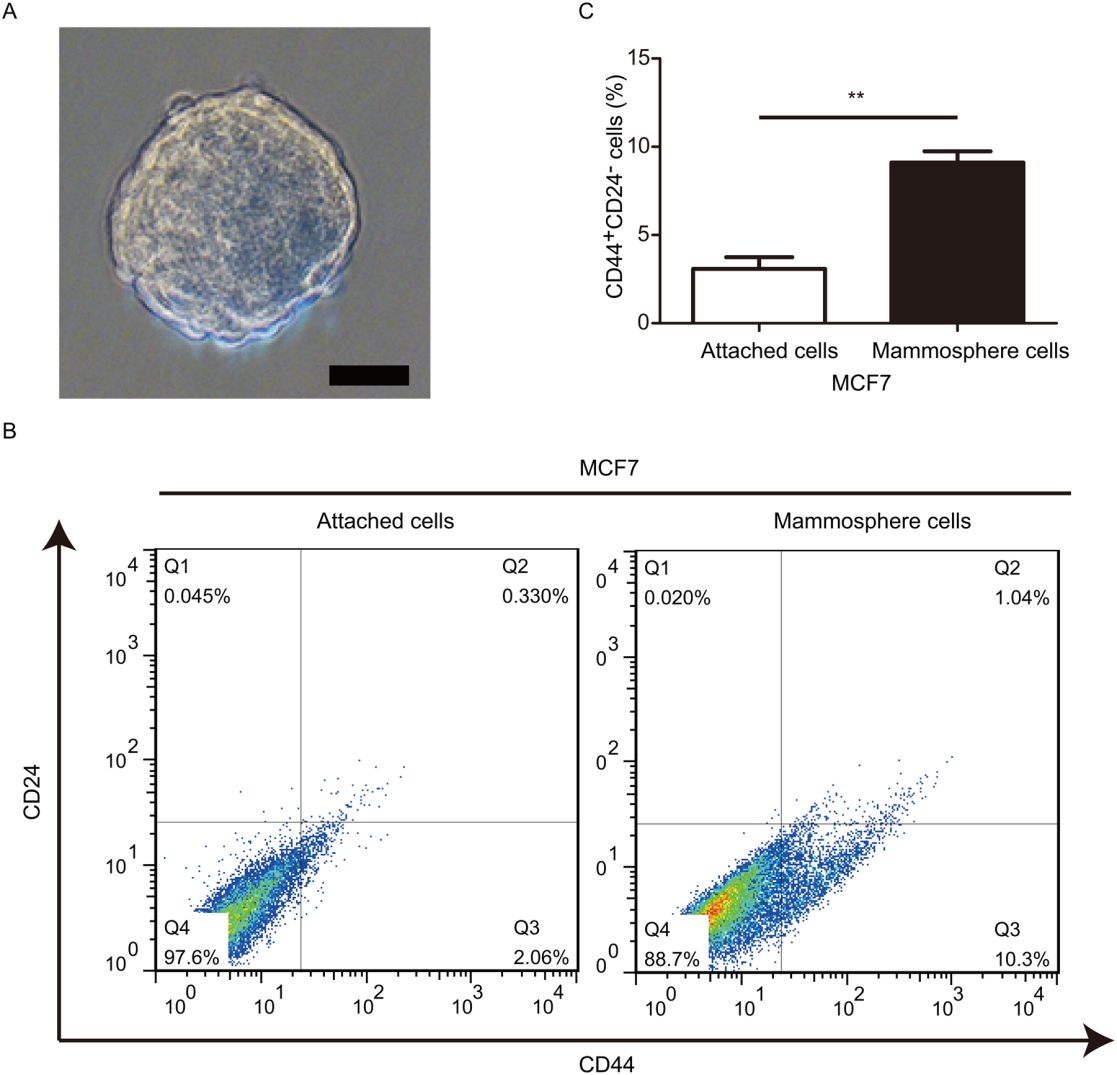
Mammosphere-derived cells exhibit a relative higher frequency of BCSCs **(A)** Phase-contrast images of mammospheres generated by MCF-7. **(B** and **C)** FACS were used to evaluate the proportions of cells with CD44^+^CD24^-^ phenotype. ^**^P< 0.01, data are represented as mean ± SD from three different assays, Scale bar, 100 *μ*m.

Mammosphere formation assay is a generally used method to obtain BCSCs *in vitro* [26]. Hence, here we conducted mammosphere assay to enrich BCSCs. Mammospheres with diameter≥ 100 *μ*m were considered as significant (Figure 1A). In the current study, our data suggested that BCSCs with CD44^+^CD24^-^ phenotye derived from mammosphere cells displayed a much higher frequency when compared to that from adherent-cultured cells (Figure 1B and 1C). These findings were in consistent with our previous report [27]. Taken together, these data validated that our platform was feasible and strengthened the notion that mammospheres were enriched CSCs.

### RNA-Sequencing reveals dysregulated circRNA signature in BCSCs

**Figure 2:**
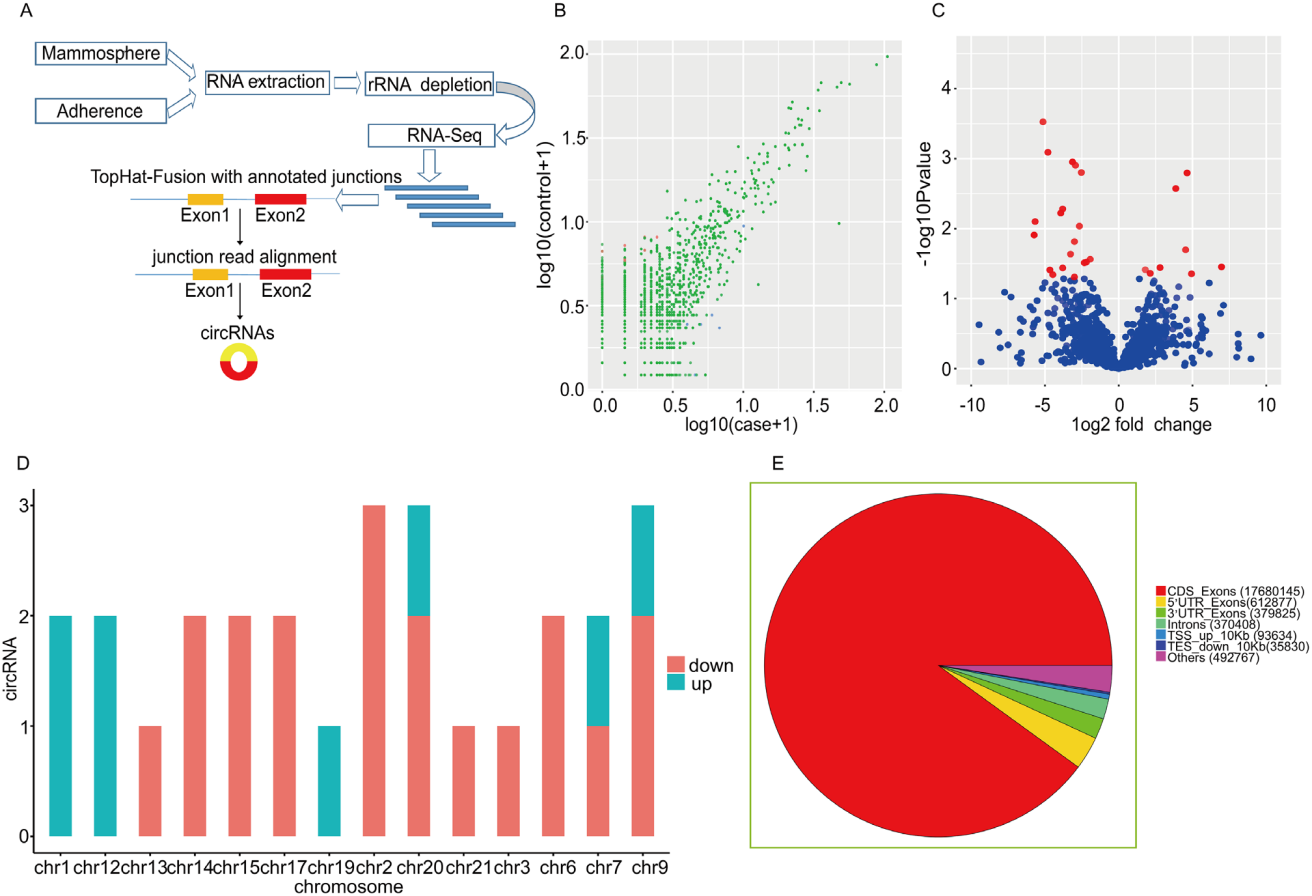
Discrepancy and characterizations in circRNA expression signature between BCSCs and non-BCSCs **(A)** Flow chart of circRNA detection and annotation. **(B)** The difference in the expression of circRNAs between BCSCs and non-BCSCs was estimated with the scattered plot. The values drawn on X and Y axes were the standardized signal values of each group (log10 scaled). Red dots indicated downregulated circRNAs and blue ones represented upregulated circRNAs. **(C)** Volcano plots were depicted to assess the differential expression between the 2 groups. The Y axis showed a *P*-value of 0.05 (−log10 scaled). The horizontal line equaled 2.0 fold (log10 scaled) up and down, respectively. The red dots in the plot indicated the aberrantly expressed circRNAs with statistical significance. **(D)** The distribution of aberrantly expressed circRNAs in human chromosomes. **(E)** The pie diagram showed the circRNA category. Most of the differentially expressed circRNAs originated from the exons. Some were from introns, while a few were from other sources.

**Figure 3:**
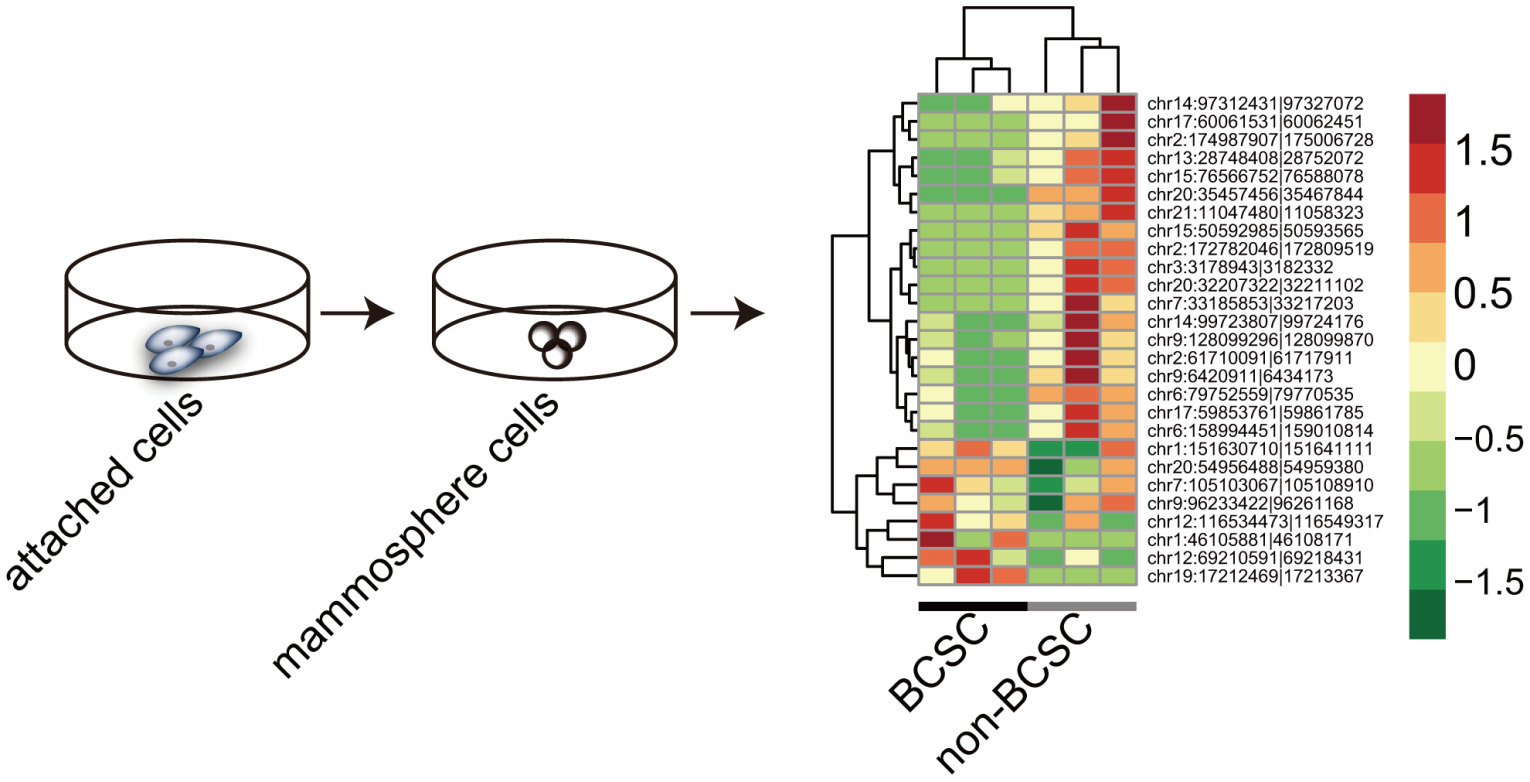
Hierarchical clusters of aberrantly expressed circRNAs between BCSCs and non-BCSCs

To investigate the expression pattern of circRNAs in BCSCs, we performed High-Throughput Sequencing to screen the expression profile of circRNAs in BCSCs and matched non-BCSCs. The flow chart was depicted in Figure 2A. A total of 5727 circRNA candidates were identified via high-throughput sequencing in 3 couple of samples. The distribution of genome alignment counts was showed in Supplementary Figure 1. Normalized log2 scales were used in scatter plot to evaluate the variations between two groups (Figure 2B) and the dysregulated circRNAs identified here were displayed in the volcano plot (Figure 2C). To further depict the traits of circRNAs, the distribution of circRNAs on human chromosomes were analysed (Figure 2D). In line with previous reports, the current study showed that circRNAs were derived from exons in most cases, some circRNAs generated from introns were also present and other sources of circRNAs were detected (Figure 2E). We summarized the differentially expressed circRNAs information in Table 1 by fold change. In the present study, 27 differently expressed circRNAs were identified by fold change≥ 1.8, *P* value < 0.05, including 8 upregulated circRNAs and 19 downregulated circRNAs (Figure 3). These data together showed that there was a difference in the expression levels of circRNAs between BCSCs and non-BCSCs.

**Table 1:** The list of differentially expressed circRNAs

CircID	Isoform Name	Uniprot Gene name	logFC	P.Value
chr1:151630710|151641111	NM_030918	SNX27	2.133333	0.043621
chr12:116534473|116549317	NM_015335	MED13L	4.933333	0.044239
chr12:69210591|69218431	NM_002392	MDM2	6.966667	0.035245
chr13:28748408|28752072	NM_175854	PAN3	-4.8	0.000812
chr1:46105881|46108171	NM_021639	GPBP1L1	1.8	0.03871
chr14:97312431|97327072	NM_003384	VRK1	-2.33333	0.030737
chr14:99723807|99724176	NM_022898	BCL11B	-5.73333	0.012355
chr15:50592985|50593565	NM_005254	GABPB1	-2.66667	0.009209
chr15:76566752|76588078	NM_000126	ETFA	-3.8	0.005233
chr17:59853761|59861785	NM_032043	BRIP1	-2.2	0.030266
chr17:60061531|60062451	NM_005121	MED13	-3	0.01532
chr19:17212469|17213367	NM_001130065	MYO9B	4.533333	0.020082
chr20:32207322|32211102	NM_001032999	CBFA2T2	-3.93333	0.006012
chr20:35457456|35467844	NM_199181	SOGA1	-1.93333	0.0274
chr20:54956488|54959380	NM_003600	AURKA	3.866667	0.002675
chr21:11047480|11058323	NM_182481	NA	-2.53333	0.001577
chr2:172782046|172809519	NM_003642	HAT1	-2.93333	0.001248
chr2:174987907|175006728	NM_001011708	OLA1	-3.13333	0.001115
chr2:61710091|61717911	NM_003400	XPO1	-4.46667	0.045581
chr3:3178943|3182332	NM_182916	TRNT1	-5.13333	0.000298
chr6:158994451|159010814	NM_020823	TMEM181	-5.66667	0.007929
chr6:79752559|79770535	NM_017934	PHIP	-3	0.049127
chr7:105103067|105108910	NM_019042	PUS7	2.8	0.036061
chr7:33185853|33217203	NM_001033604	BBS9	-3.26667	0.02315
chr9:128099296|128099870	NM_015635	GAPVD1	-4.66667	0.038967
chr9:6420911|6434173	NM_152896	UHRF2	-3.8	0.036463
chr9:96233422|96261168	NM_014612	FAM120A	4.633333	0.001599

### Q-PCR results of six randomly selected circRNAs validate the sequencing data's reliability

**Figure 4:**
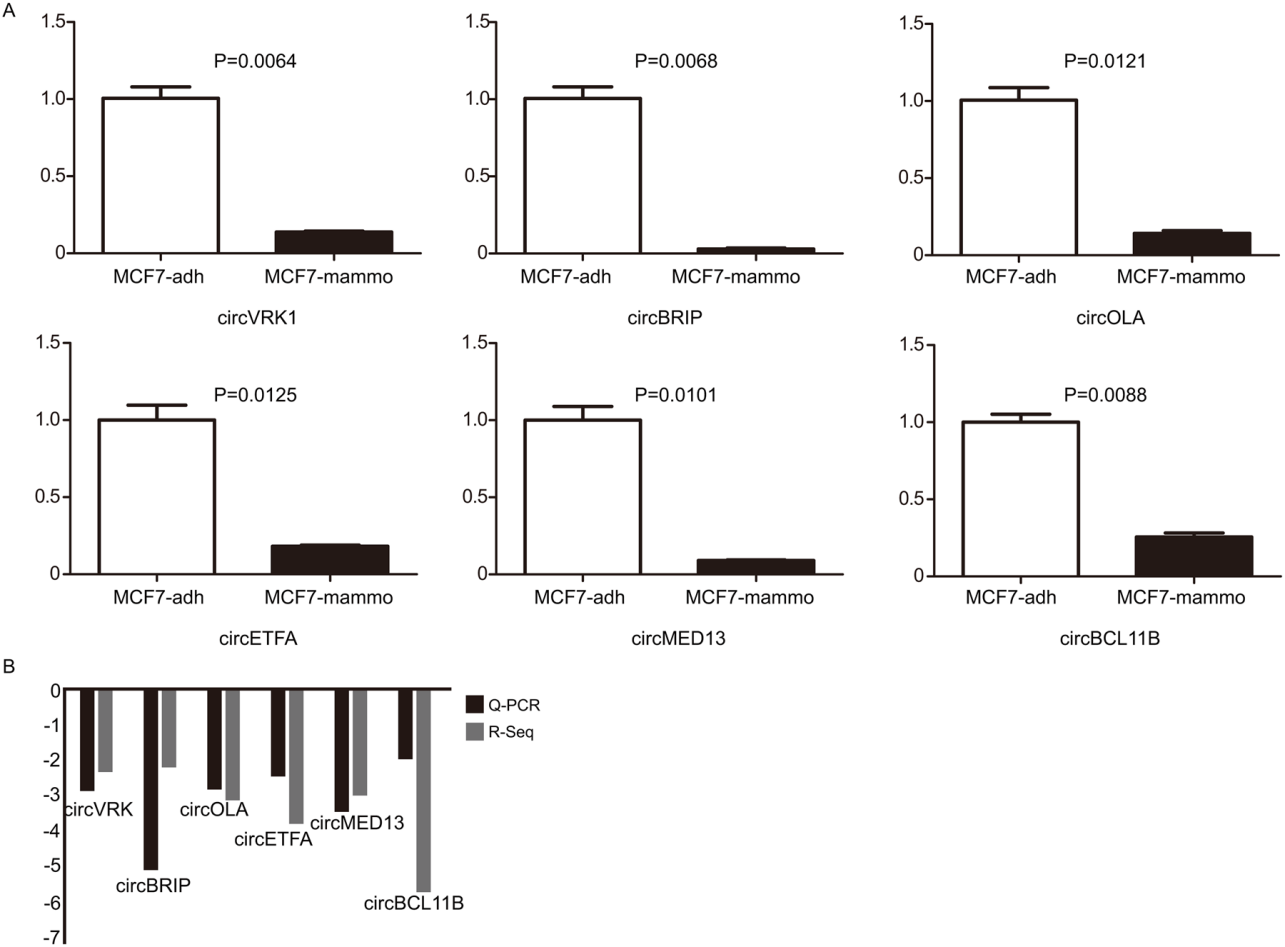
Q-PCR validates the expression of 6 selected circRNAs **(A)** The expression levels of 6 circRNAs were validated by Q-PCR in adherent cells and floating mammosphere cells. **(B)** The comparison between RNA-Seq data and Q-PCR results. The Y axis showed the average of fold change (log2 transformed) of each circRNAs measured by Q-PCR and sequencing respectively. ^*^P<0.05, ^**^P<0.01 and ^***^P<0.001, data are shown as mean ± SEM of triplicated experiments.

**Figure 5:**
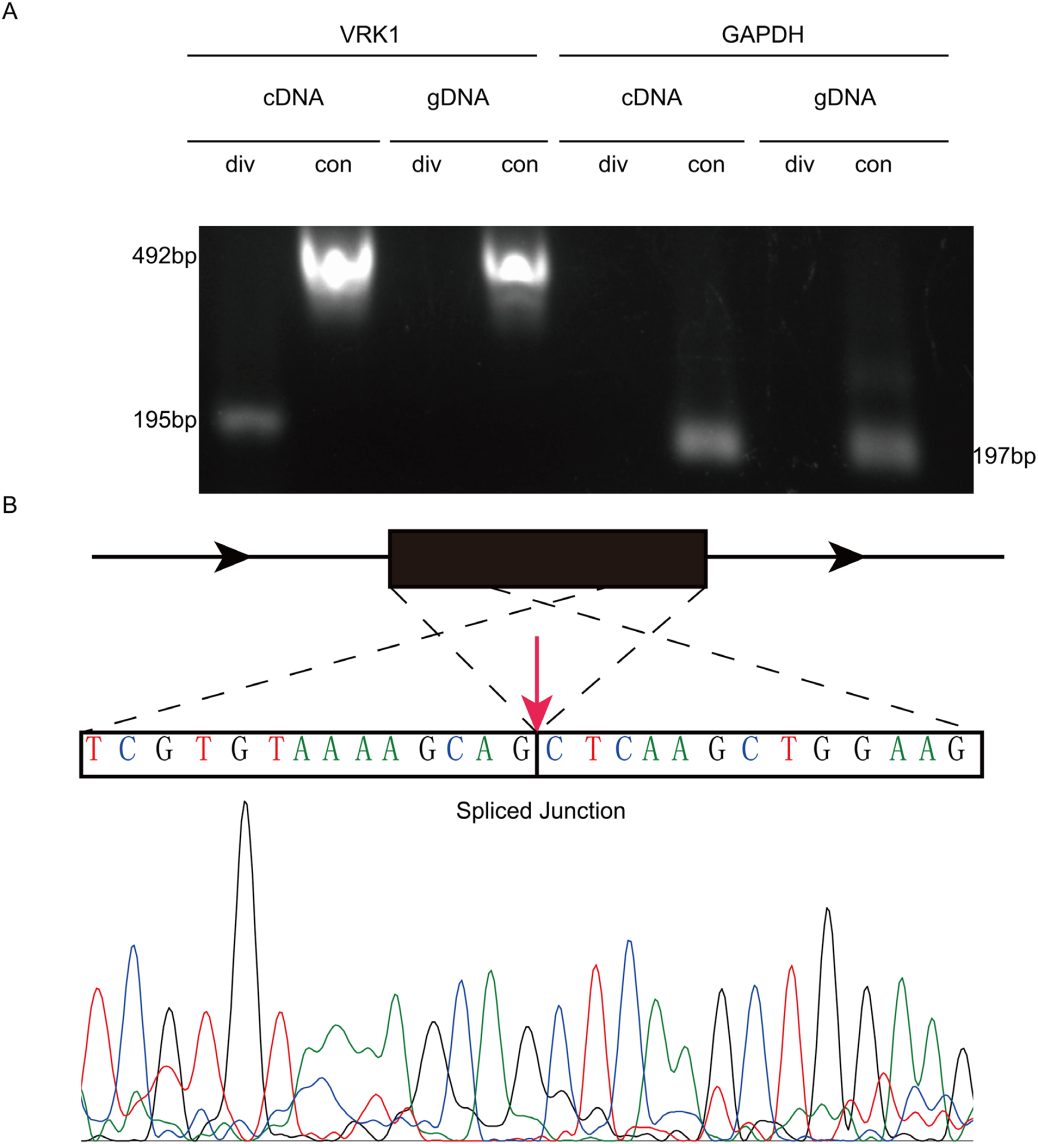
PCR and Sanger sequencing validates the existence of *circVRK1* **(A)** Gel electrophoresis, **(B)** Sanger sequencing results.

Six circRNAs were randomly selected to validate the RNA-Sequencing results via Q-PCR. Our results demonstrated that the 6 downregulated circRNAs identified by sequencing were truly downregulated in mammosphere cells compared to attached cells (Figure 4A). In addition, the log_2_ fold-changes suggested that the Q-PCR results were in agreement with RNA-Sequencing data (Figure 4B). Specifically, one of selected circRNAs, *circVRK1* were further confirmed with sanger sequencing. Our data showed that only cDNA templates possessed the potential to amplify *circVRK1*, however, both cDNA templates and gDNA templates were able to obtain linear *VRK1* (Figure 5A and 5B). These results showed that RNA-Sequecing analysis was in line with Q-PCR results and indicated that these circRNAs were significantly differentially expressed.

### The potential functionalities of identified circRNAs are predicted via GO and KEGG pathway analysis

**Figure 6:**
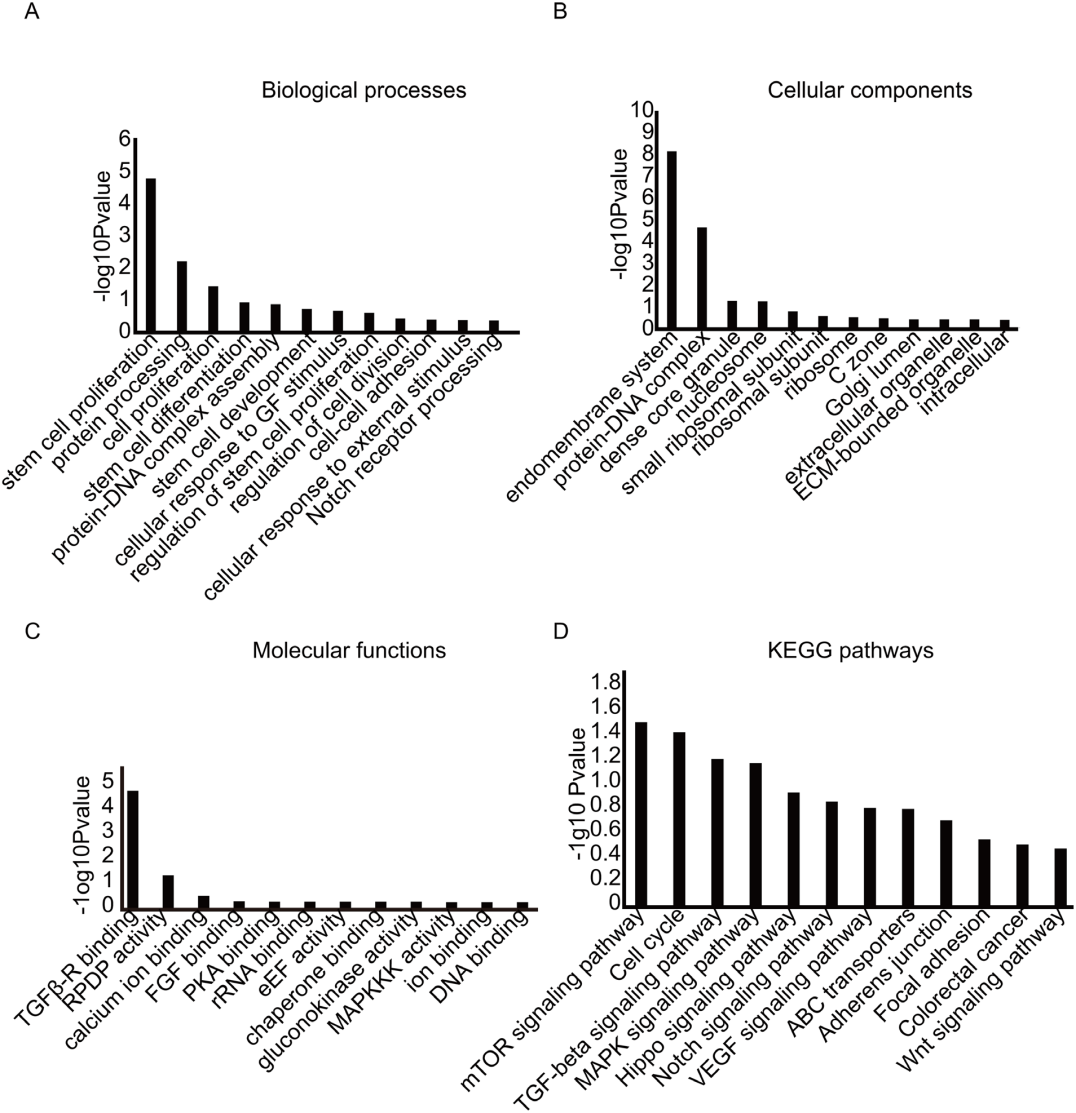
GO and KEGG pathway analysis The GO database includes 3 parts. **(A)** biological process, **(B)** cellular component, **(C)** molecular function, **(D)** KEGG pathway analysis.

To investigate the functional roles of identified circRNAs, GO enrichment analysis and KEGG pathway analysis were employed to analyse the functional effects of dysregulated circRNAs. GO analysis indicated that the differentially expressed circRNAs were present predominantly in the organelle of the endomembrane system, such as the ribosomes, and lumen of the Golgi (Figure 6A). Further, GO analysis showed that biological processes including stem cell proliferation, differentiation, and development were correlated with the identified dysregulated circRNAs, which suggested that the differentially expressed circRNAs identified here were possible to be involved in biogenesis of BCSCs (Figure 6B and 6C). KEGG pathway analysis for the circRNAs showed strong enrichment for stem-cell-characteristic-related pathways including the Hippo signaling pathway, Notch signaling pathway, and Wnt signaling pathway. To date, abundance of reports have revealed that these pathways were strongly linked with stem cell characteristics [28–30]. The particulars of functional analyses were summarized in Figure 6D. These results taken together provided new viewpoints on the functionalities of circRNAs on stemness of BCSCs.

### CircRNA/miRNA interaction network shows that the screened circRNAs are possible to serve as miRNA sponges

**Figure 7:**
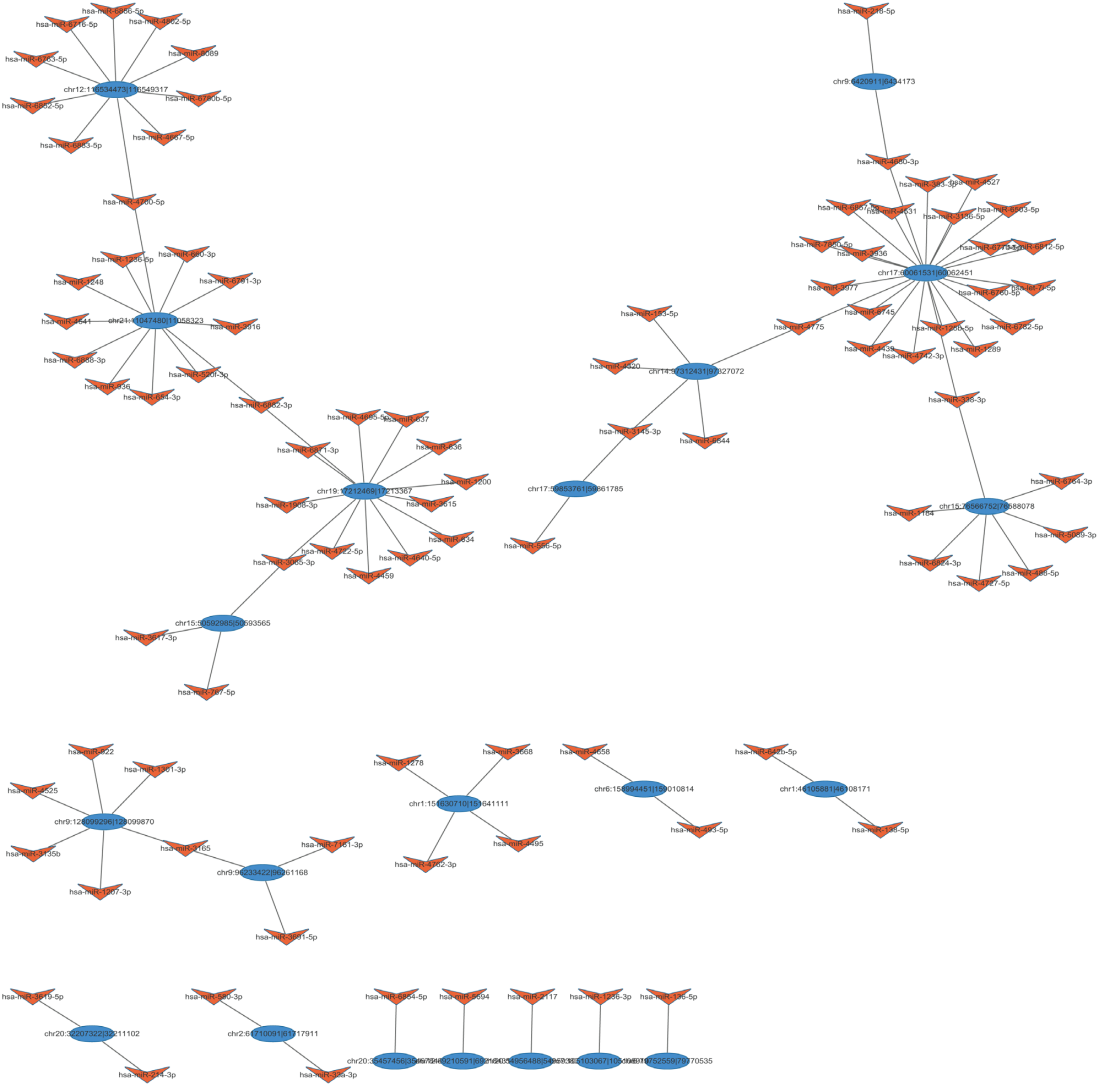
The circRNA/miRNA network analysis The full view network consists of the differentially expressed circRNAs (blue) and their target miRNAs (orange).

Reports suggested that circRNAs might binding with miRNAs thus regulating gene expression as miRNA sponges [31]. Hence, miRANDA and TargetScan were using to predict miRNA targets per conserved seed-matching sequences. In the present data, a total of 2712 miRNA candidates were identified (data not shown). To better delineate the interactions, the top 103 target miRNAs and their counterpart circRNAs were chose to construct the interaction network using cytoscape (Tot. Score≥ 90, Tot. Energy≤ –17) (Figure 7). Of these, we found that mir-153-5p was one of the predicted miRNA targets of *circVRK1*, which was one of the downregulated circRNAs in BCSCs. Interestingly, a previous study revealed that mir-153 was involved in stemness maintenance of triple-negative breast cancer via reducing the expression of KLF5 [13]. Combined these results with our data, these findings prompted us to hypothesize that *circVRK1* was possible to negatively correlated with stemness of BCSCs.

### CircVRK1 displays an inhibiting role in stemness of BCSCs

**Figure 8:**
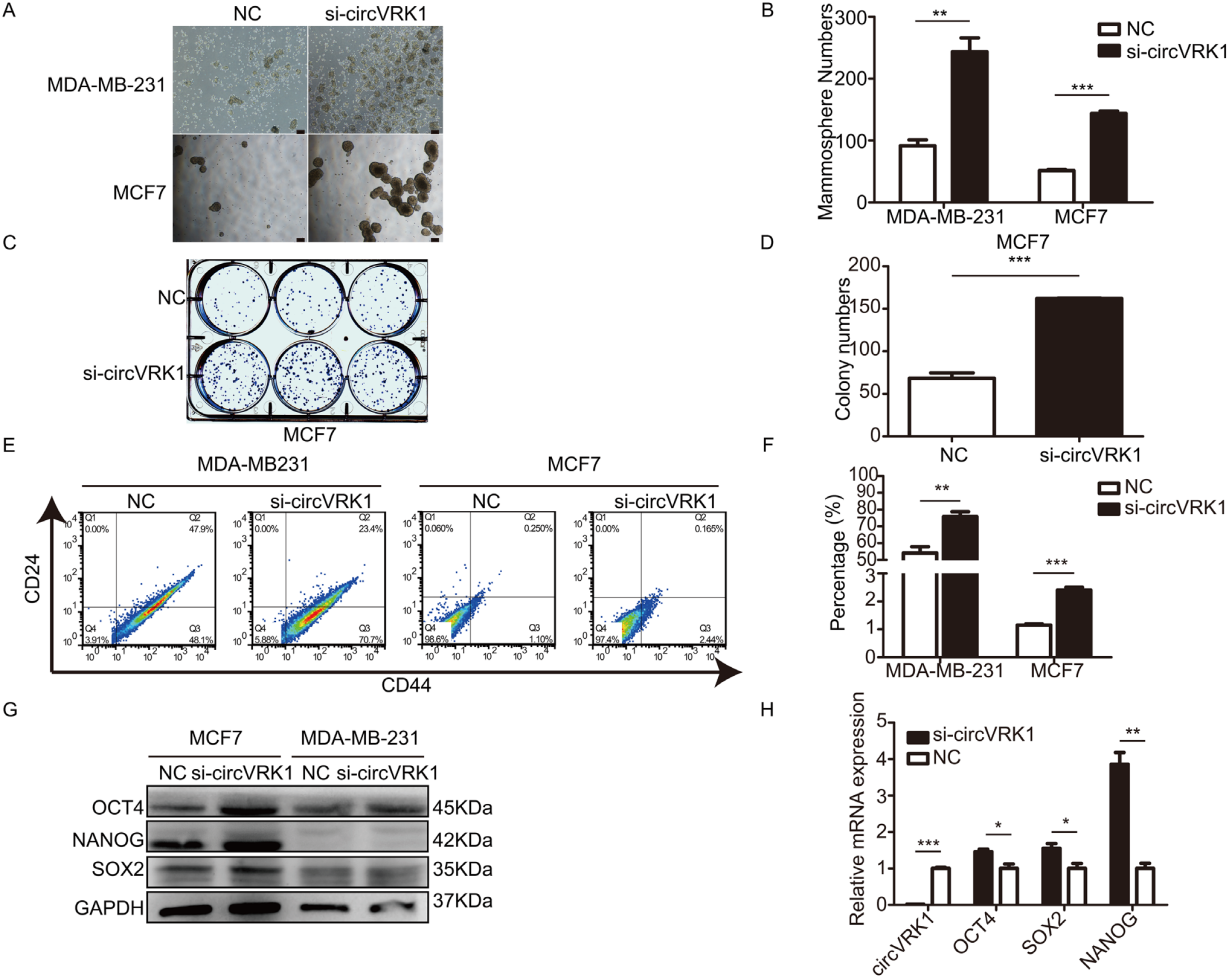
*CircVRK1* displays a negative regulatory effect on self-renewal capacity, BCSCs’ expansion and expression levels of stemness-related markers *in vitro* **(A** and **B)** Size and numbers of mammospheres derived from breast cancer cells treated or not with *si-circVRK1*. **(C** and **D)** Size and numbers of colonies cultured from breast cancer cells treated or not with *si-circVRK1*. **(E** and **F)** Proportions of CD44^+^CD24^-^ in breast cancer cells were increased when treated with si*-circVRK1* compared to NC. **(G** and **H)** The global abundance of stemness-related markers were markedly increased when treated with *si-circVRK1* not only at mRNA level but also protein level. All the results were obtained from three independent experiments and the data are reported as mean ± SD. ^*^P<0.05, ^**^P<0.01 and ^***^P<0.001, Scale bar, 100 *μ*m.

To testify our hypothesis, we designed siRNAs to silence *circVRK1* and explored its influence on self-renewal capacity, BCSCs’ expansion and expression of stemness-related markers. We found that breast cancer cells showed an enhanced capacities to form mammospheres and colonies after loss of *circVRK1* (Figure 8A–8D). Additionally, the proportion of BCSCs with CD44^+^CD24^-^ phenotype were significantly increased when reduced *circVRK1* (Figure 8E and 8F). Similarly results were also observed in the global level of stemness-related factors (Figure 8G and 8H). Taken together, these data indicated that *circVRK1* was negatively correlated with maintenance of BCSC characteristics.

## DISCUSSION

CircRNAs, an enigmatic type of RNAs, were first identified as aberrantly spliced transcripts in eukaryotes. Since its first discovery in 20 years ago, only a handful of circRNAs were detected, and they were frequently considered as noises or artifacts of abnormal splicing with little function [32–35]. Recently, with the development of bioinformatic approaches and sequencing technology, the abundance of circRNAs were found widespread in organisms [17, 31, 36, 37]. Although an increasing number of circRNAs were found continuously, the functional roles of circRNAs in CSCs were poorly studied.

In the present study, we were the first to report on the circRNA repertoire in BCSCs. There have been some lines of research into circRNA profile in cancer [22, 23, 38, 39], however, to the best of our knowledge, circRNA signature has never been explored in CSCs. Our results revealed that 27 circRNAs were differentially expressed, of which 19 circRNAs were downregulated and 8 were upregulated relative to the non-BCSCs (Figure 3). Moreover, all 27 identified circRNAs were aberrantly expressed with fold change> 1.8, which lent strong supports that these circRNAs were correlated with BCSCs. To testify the reliability of RNA-Sequencing results, six circRNAs, *circVRK1*, *circBRIP*, *circOLA*, *circETFA*, *circMED13* and *circBCL11B*, were randomly selected to make the validation via Q-PCR. Our results indicated that all the six circRNAs were downregulated in mammosphere-derived cells compared with adherent cells *in vitro*, which meant that Q-PCR results were consistent with the RNA-Sequencing data (Figure 4).

Recently, a large body of evidence has revealed that circRNAs were able to regulate the expression of their parent genes [39–41]. Hence, the functions of mRNAs were possible to mirror the roles of corresponding circRNAs. Therefore, GO and KEGG pathway analysis were conducted to evaluate functional roles of circRNAs in BCSCs. GO analysis for the differential expressed circRNAs revealed that some terms under the biological process and molecular function categories were associated with the stem cell characteristics (Figure 6A–6C). These findings indicated that circRNAs revealed by RNA-Sequncing were mostly expressed in the cytoplasm. A recent report have reported that plenty of circRNAs appeared to be specifically present in developmental stage or cell-specific manner and confirmed that CDR1as was highly expressed in cytoplasm [17]. Hence, our work was partially in accordance with the report. Additionally, KEGG pathway analysis identified plenty of pathways associated with the stemness of stem cells including Hippo pathway [28, 42], Notch pathway [29], Wnt pathway [30], and TGF-β pathway [43] (Figure 6D). Combined with the current work, it was easy to conclude that the differentially expressed circRNAs might be implicated in sustaining the self-renewal and multipotent capacities of BCSCs. Hence, determining the function of the detected circRNAs could be beneficial for radical elimination of BCSCs.

In addition, circRNA/miRNA interplay network revealed here provided the first reported evidence to investigate the functional roles of circRNAs in BCSCs. The present work demonstrated that most of 27 aberrantly expressed circRNAs could interact with one or more miRNAs via bioinformatics analysis. Specifically, we found that mir-153 was one of the target miRNAs of *circVRK1* (Figure 7). Interestingly, a recent published study revealed that mifepristone could inhibited the expression of KLF via inducing the expression of mir-153, thus leading to suppression on stemness of triple negtive breast CSCs [13]. These results indicated that *circVRK1* might be implicated in inhibiting stemness of BCSCs through adsorbing mir-153. Previous studies revealed that circRNAs were able to serve as Competitive endogenous RNAs (ceRNAs) or miRNA sponges, which controlled the expression levels of the target genes via competing with miRNAs for binding sites [17, 18]. For instance, cerebellar degeneration related 1 antisense transcript (CDR1as), a well-studied circRNA, was proven to harbour more than 60 conserved miR-7 binding sites which were common in humans and other species [17, 18]. CDR1as was dominantly expressed in cytoplasm and might bind up to 20,000 miR-7 molecules per cell due to CDR1as and miR-7 shared specific expression domains [17]. Once CDR1as binding with miR-7, the power of miR-7 to regulate its downstream target genes was drastically attenuated. Besides, testis-specific circRNA, sex-determining region Y (Sry) was also found to harbour 16 miRNA binding sites for its target miR-138 in mice, although only one target site was found to have a human homologue [18]. Additionally, recent studies have shown that circ-ITCH could serve as a miRNA sponge by binding with miR-7 and miR-124. In this fashion, circ-ITCH could upregulate the expression of its parent gene ITCH [39]. In principle, any RNA transcripts with MREs could act as ceRNAs to sequester miRNAs. Disrupting the equilibrium among ceRNAs was found to be capable of triggering malignant biological events. However, it was noteworthy that there has been no convincing evidence supporting the conclusion that all known circRNAs could act as miRNA sponges. On the contrary, most human and mouse circRNAs seemed to have very few miRNA target sites, suggesting they might not function as miRNA sponges [44]. Hence, our results remained further investigation.

According to the BCSCs theory, BCSCs were defined by their properties, including increasing self-renewal, multilineage differentiation, and resistance to chemotherapy, all of which were considered as contributing to the breast cancer progression, metastasis and recurrence [45, 46]. The first line of evidence for the existence of BCSCs demonstrated that a celluar population with CD44^+^CD24^-^ phenotype displayed the capacity to initiate new tumours in NOD/SCID Mice [47]. In addition, BCSCs exhibited the ability to form mammospheres, which was commonly used method to enrich BCSCs *in vitro*. Hence, inhibiting these characteristics was an effective way to kill BCSCs. Therefore, to investigate the functionality of *circVRK1* on BCSCs, we silenced *circVRK1* with siRNA targeting *circVRK1* and observed its implication in BCSC behaviors. We found that the breast cancer cells displayed an increased capacity of self-renewal and expanded BCSCs’ expansion when loss of *circVRK1* (Figure 8A–8F). In addition, an increasing global levels of stemness-related markers including NANOG, SOX2 and OCT4 were observed when *circVRK1* was silenced (Figure 8G and 8H). these data taken together provided strong supports that *circVRK1* were implicated in suppressing BCSCs. Honestly, the current work remains further investigation and future studies should focus on the dysregulation of the *circVRK1*/miR-153 axis in BCSCs.

In conclusion, the current work was the first to indicate the circRNA signature of BCSCs. We determined the circRNA/miRNA network and analysed the potential functional roles of dysregulated circRNAs. Furthermore, we revealed that *circVRK1* was capable to negatively regulate the stemness of BCSCs. Our findings strengthen the possibility that *circVRK1* is able to serve as a potential target for BCSCs. Additionally, the current data also provide supports for further study on circRNAs in stemness maintenance of BCSCs.

## MATERIALS AND METHODS

### Antibodies

Rabbit monoclonal anti-NANOG (CAT: 4903S), anti-SOX2 (CAT: 3579S), anti-OCT4 (CAT: 2750S) and anti-GAPDH (CAT: 5174S) used for western blot assays were purchased from Cell Signaling Technology (CST, US). Goat anti-rabbit IgG-HRP was obtained from Santa Cruz Biotechnology (Santa Cruz, US). Antibodies to FITC-conjugated CD44 (CAT: 130-095-195) and PE-conjugated CD24 (CAT: 130-095-953) used for Fluorescence-activated cell sorting (FACS) were ordered from Miltenyi Biotec (US).

### Cell culture

The human breast cancer cell line MCF-7 and MDA-MB-231 were obtained from American Type Culture Collection (ATCC) and were maintained in recommended media according to the ATCC's instructions. Basically, they were cultured in DMEM high glucose media supplemented with 10% fetal bovine serum (FBS) (Hyclone, US), 100 U/ml penicillin and 100 μg/ml streptomycin (Invitrogen, US) at 37 °C.

### Mammosphere formation assay

Mammosphere formation assay was performed as described previously [48]. In brief, single-cell suspensions at a density of 1×10^4^/ml were inoculated in a ultra-low attachment six well plate (corning, US) and maintained in serum-free DMEM/F-12 (Hyclone, US) supplemented with 50×B27 (Invitrogen, US), 20 ng/ml human epidermal growth factor (EGF) (Gibco, US), 20 ng/ml human basic fibroblast growth factor (h-basic-FGF) (Gibco, US) and 5 μg/ml human insulin (Sigma-Aldrich, US), then cultured in an incubator at 37°C with 5% CO_2_. The medium was replaced every 3 days and mammospheres were collected at day 7.

### Colony formation assay

Breast cancer cells were digested and resuspended at a density of 10^4^/ml. Thereafter, 600 cells were plated into a six well plate, and were maintained in medium and monitored for colony formation. At day 14, the clones were confirmed with microscope and clones more than 50 cells were considered as significant.

### FACS analysis

The mammosphere-cells were detached by trypsin and resuspended in 1×PBS supplemented with 2.5% FBS, then incubated with FITC mouse anti-human CD44 and PE mouse anti-human CD24 in staining buffer including 1% bovine serum albumin and 2 mM ethylene diamine tetraacetic acid for 10 min at 4°C. Following, stained cells were subjected to sorting and divided to 2 groups: CD44^+^CD24^-^ cells (BCSCs) and non- CD44^+^CD24^-^ cells (non-BCSCs). Data were analysed with a FACS Aria (BD Biosciences).

### Total RNA extraction and Rnase R digestion

Total RNAs were extracted from BCSCs and non-BCSCs in concordance with the manufacturers’ protocol. RNA quantity and quality was evaluated by Nano Drop ND-1000 spectrophotometer (Thermo Fisher Scientific, US). In addition, the RNAs were subjected to agarose gel electrophoresis to estimate its integrity. Then the RNAs were subjected to ribosomal RNA (rRNA) depletion with ribo-zero-magnetic-kit according to the instructions. RNase R treatment was carried out as established previously [40]. Simply, processed RNAs were incubated using RNase R (Epicenter) for 3 h at 37°C to remove the linear RNAs.

### Detection of circRNAs

CIRCexplore was carried out to detect circRNAs in BCSCs and non-BCSCs samples as described previously [37]. Briefly, 2-step mapping strategy was performed. Firstly, TopHat was used to perform the multiple mapping to the sequence reads of all the samples. Then unmapped reads were screened and mapped to reference genome (GRChr37/hg19) using TopHat-Fusion [49]. These reads were separated and mapped onto the relevant reference genome while non-linear candidate positions were considered possible back-splice junction reads. Next the candidate reads were further matched to the existing gene annotation to confirm the exact splice location, the donor and acceptor positions. Notably, reads that aligned on different genes or non-canonical splice sites were mostly considered as artifacts of trans-splicing or PCR errors, these reads were not used as candidates.

### Characterization of circRNAs

The subsequent analysis was performed to fully delineate the characteristics of identified circRNAs. Firstly, the distribution of genome alignment counts in chromosomes was analysed. Secondly, the difference of circRNAs between BCSCs and non-BCSCs was evaluated. Thirdly, the distribution of dysregulated circRNAs in chromosomes was determined and the sources of aberrantly circRNAs between BCSCs and non-BCSCs were analysed. In addition, Hierarchical cluster analysis was applied to depict the differentially expressed circRNAs.

### Construction of circRNA/miRNA interplay network

Studies have shown that circRNAs could act as miRNA sponges [17, 18]. Hence, experiments were performed to determine whether the aberrantly expressed circRNAs identified here had the potential to interact with miRNAs. Possible miRNA targets were predicted using miRanda and TargetScan database. To construct circRNA-miRNA network, circRNAs possessing MREs were screened in Tot. Score≥ 90 and Tot. Energy≤ –17, then the miRNAs were selected in accordance with seed match sequences. The circRNA/miRNA interaction network was depicted using Cytoscape software.

### Bioinformatics analysis

To investigate the potential functions of the differentially expressed genes, GO enrichment analysis was consulted to describe gene and gene products attributes (http://www.geneontology.org). Distinct GO subtypes were considered significantly enriched when *P*-values< 0.05. KEGG pathway analysis was conducted to determine aberrantly genes in different biological processes (http://www.kegg.jp). The *P*-value here represents the significance of the pathways. The lower the *P*-value, the more significant the pathway.

### Q-PCR

Reverse transcription for circRNAs was implemented using Super-Script II (Takara, Japan) as per the manufacturers’ instructions. Q-PCR was performed with a 1:10 dilution of the cDNAs with FastStart Universal SYBR Green Master Kit (Roche, Switzerland) and an ABI PRISM 7900HT sequence detection system was applied to execute the program (Applied Biosystems, U.S.). Two pairs of primers (convergent primers and divergent primers) were designed for each selected circRNA by primer 5 and are given in Supplementary Table 1. Theoretically, convergent primers were supposed to amplify the linear transcripts, while divergent primers were capable of achieving the circular amplification products. CDNAs transcribed from total RNAs served as templates, and genomic DNAs (gDNAs) were selected as a control. The RT-PCR protocol was as follows: first, 2 min at 50°C, followed by 10 min at 95°C, next to 40 cycles of PCR followed standard conditions with 15 s denaturation at 95°C, elongation at 60°C for 1 min, then at 95°C for 15 s, and 1 min for 60°C. The relative abundance was normalized to GAPDH, and fold change was analysed with the 2^-ΔΔCt^ method [50]. Error bars represent standard deviations. All the experiments were performed in triplicate.

### Western blotting

Breast cancer cells were harvested and lysed in RIPA lysis and extraction buffer (Thermo Fisher Scientific, US), Western blotting was carried out as previously described [27]. Briefly, equal amounts of proteins were loaded into SDS-PAGE gels and transferred onto polyvinylidene difluoride membrane (Millipore, US). Then the membranes were blocked with 5% non-fat milk for 1 h at room temperature, and incubated with primary antibodies overnight at 4°C. Then membranes were washed with 1×PBST and incubated with horseradish peroxidase-conjugated secondary antibodies (Santa cruz, US) for 1 h. Images were obtained through a LAS-3000 Imager (Fuji film).

### Sanger sequencing

PCR was carried out for 40 cycles according to the protocol. Detailed, denaturation at 95°C for 30 s, followed by annealing at 60°C for 30 s, then elongation at 72°C for 1 min. PCR products were resolved and size separated on 1.5% agarose gel supplemented with yealRed Nucleic Acid Gel stain (Yeasen, China) and confirmed by Sanger sequencing with standard methods (Genewiz, China).

### Silencing of circVRK1

Breast cancer cells were transfected with a siRNA target *circVRK1* or Negative Control (NC) (GenePharma, China) using Lipofectamine 2000 (Invitrogen, US), after treated for 48 h, cells were collected for further research. The siRNA sequence for *circVRK1* and NC were as follows:

*CircVRK1*: Sense: 5’ - GCAGUUGGAGAGAUA AUAATT,

Antisense: 5’ -UUAUUAUCUCUCCAACUGCTT.

NC: sense: 5’ – UUCUCCGAACGUGUCACGUTT,

Antisense: 5’ – ACGUGACACGUUCGGAGAATT.

### Statistical analysis

All data were analysed using Prism 5 unless where indicated (GraphPad Software, Inc.). Student's t-test was used for the comparison in the aberrant levels of expression of circRNAs between distinct groups. Fisher's exact test was performed in GO and KEGG pathway analysis. The mean ± SEM was used to evaluate the values. All experiments were performed in triplicate, and *P*-values< 0.05 were considered statistically significant.

## SUPPLEMENTARY MATERIALS FIGURES AND TABLES



## Abbreviations

BCSCs: breast cancer stem cells
CSCs: cancer stem cells
ncRNAs: non coding RNAs
MRE: micro RNA response element
circRNAs: circular RNAs
EMT: epithelial mesenchymal transition
Q-PCR: Quantitative PCR
miRNA: microRNA
ATCC: American Type Culture Collection
EGF: human epidermal growth factor
h-basic-FGF: human basic fibroblast growth factor
rRNA: ribosomal RNA
GO: Gene Ontology
KEGG: Kyoto Encyclopedia of Genes and Genomes
NC: negative control
ceRNAs: Competitive endogenous RNAs
CDR1as: cerebellar degeneration related 1 antisense transcript
Sry: sex-determining region Y
