# Supplementary material for: Circular RNA profile indicates circular RNA VRK1 is negatively related with breast cancer stem cells
Source: Oncotarget. 2017 Sep 23;8(56):95704–18. doi: 10.18632/oncotarget.21183 (PMC5707054; doi:10.18632/oncotarget.21183)
Supplement: Supplementary file 1 [file oncotarget-08-95704-s001.pdf]

Circular RNA profile indicates circular RNA VRK1 is negatively related with breast cancer stem cells

SUPPLEMENTARY MATERIALS

Supplementary Table 1: The list of primers

Gene	Primers	Linear/Circular	*Sapiens*
VRK1	Forward: GAACCTGGTGTTGAAGATACGG<br>Reverse : AATCCTACTTTCCATTCCTTTTTTG	Circular	*Homo*
BRIP	Forward: TCTTATTCTAGTGGATGATCGCTTT<br>Reverse : GAAGGTGGTGTGCTTGGATAGTT	Circular	*Homo*
OLA1	Forward: AATGGTCTACTTGGTTAATCTTTCTG<br>Reverse : TTTTATCTCCTCCTCTCACAGCC	Circular	*Homo*
ETFA	Forward: CAGATGCCAGAATGCTTGTTTAT<br>Reverse : GTTCCAGCTACTAAGCAGGACACT	Circular	*Homo*
MED13L	Forward: ATCGGCTACTATCAACAGAACCTC<br>Reverse : GAGTAGGAATGTCTGACTGAGGGA	Circular	*Homo*
BCL11B	Forward: ACGAAAGGCATCTGTCCCAA<br>Reverse : TTGTGCTCTATAAAAACCAGGATGT	Circular	*Homo*
GAPDH	Forward: TCCTCACAGTTGCCATGTAGACCC<br>Reverse : GCGGGCTCAATTTATAGAAACCGGG	Circular	*Homo*
GAPDH	Forward: GGAGCGAGATCCCTCCAAAAT<br>Reverse : GGCTGTTGTCATACTTCTCATGG	Linear	*Homo*
VRK1	Forward: GCTGCCGAGTTACGAGTC<br>Reverse : AGGTCACTCCCAAAGCGATC	Linear	*Homo*

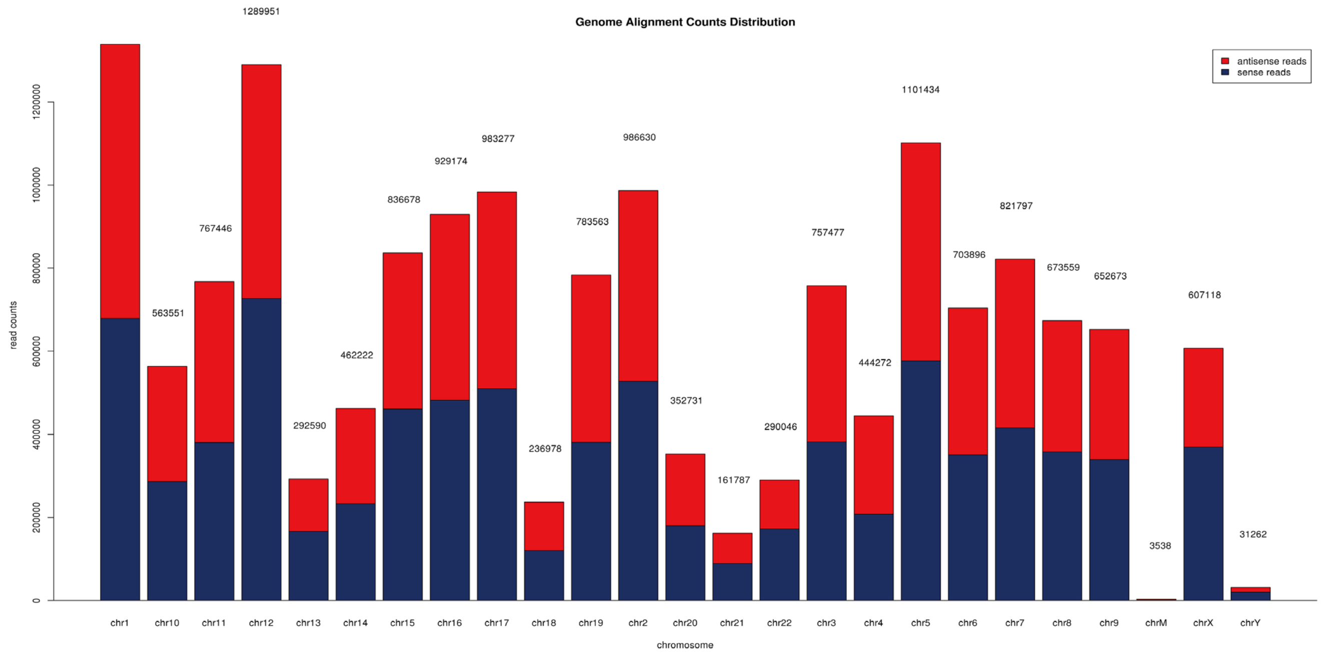

Supplementary Figure 1: The distribution of genome alignment counts.
